# Please comment, but with civility

**DOI:** 10.1186/1742-4690-5-35

**Published:** 2008-04-24

**Authors:** Kuan-Teh Jeang

**Affiliations:** 1 The National Institutes of Health, Bethesda, MD, USA

## Abstract

*Retrovirology *provides the opportunity for readers to post comments online for all published articles. These comments are moderated by the journal editors and are often peer reviewed prior to posting. *Retrovirology *welcomes comments, but asks that they be written civilly.

*Retrovirology *is an Open Access journal. Every *Retrovirology *article is immediately available upon publication for all to read online, fee-free, in full text [1,2]. An Open Access journal is different from a subscription journal because the latter requires readers to pay a fee in order to access the full text content. On April 7^th^, 2008, the United States National Institutes of Health (NIH) nudged journal publishing a step closer towards universal Open Access. Beginning on that day, NIH stipulated that all manuscripts accepted for publication from NIH funded research must be deposited in full text into PubMed Central (PMC), an NIH online digital repository. Such articles in PMC will become freely accessible for all to read without charge no later than 12 months after publication.

The NIH move is timely, and it parallels a growing trend that "fee-free" online access has become the public's choice for securing knowledge. For example, the *Wall Street Journal *reported on April 18, 2008, that print/subscription revenue at a major American newspaper, the *New York Times (NYT)*, declined by 12.5% in March 2008 compared to March 2007. Interestingly, in the same report, fee-free access to the *NYT*'s online news site was shown to have increased steadily and robustly.

*Retrovirology'*s 5-year experience echoes the above findings. It is not unusual that a published *Retrovirology *article is accessed one thousand or more times within the first week of its online appearance. In fact, during 2007, the two most highly accessed *Retrovirology *papers were each read more than 5,000 times [3]; [4]. Although we do not have firm data, elsewhere it has been shown that Open Access articles are more widely read and more highly cited than their non-Open Access counterparts [5].

Open Access publishing appears to clearly benefit authors. Interestingly, the current technology of online publishing holds similar promise for interactive and nearly instantaneous participation from readers. Unlike printed journals which cannot allow readers to quickly share their thoughts with others, *Retrovirology's *"comment" tool is a facile digital means for a reader to post his/her immediate impressions of/reactions to a published piece. A feature of Open Access is that the possible range of contributors to the comment forum becomes vastly greater than those who are permitted at a subscription site (e.g. commentators could include journalists, students, retired researchers, or anyone in the interested public).

A posted "comment" at *Retrovirology *is always moderated by an editor and is frequently peer-reviewed. It is a tad disappointing that most readers are reticent about posting "comments" at *Retrovirology*, and a similar reluctance is seen at other journals. Nevertheless, on occasion, lengthy postings appear and are followed quickly by thoughtful rejoinders from authors [6]. These lively but considered exchanges inform the discussion and enrich the dissemination and interpretation of scientific results.

Some comments proffered by readers are declined by *Retrovirology*. Without going to great lengths into individual reasons for each case, these unposted comments usually suffer from one or more of the following deficiencies: they are belittling of colleagues; they contain personal aspersions; they are oblivious to balanced views on an issue; or they lack civility. Why does civility matter? It matters because civility respects the vulnerabilities of participants, and it elevates the dialogue to focus on the content being communicated rather than the process or the inferred motives of information exchange. At *Retrovirology*, we welcome and encourage your comments, but we urge you to be civil.

Two final thoughts on posting comments – wait 24 hours and allow your emotion to cool before writing your missive; and by all means disagree, but please don't be disagreeable!
